# Racial disparities between measures of area deprivation and financial toxicity, and uterine volume in myomectomy patients

**DOI:** 10.1186/s12905-023-02761-x

**Published:** 2023-11-14

**Authors:** Andrew S. Bossick, Joelle Aoun Abood, Ashlee Oaks, Annmarie Vilkins, Ghadear Shukr, Petra Chamseddine, Ganesa R. Wegienka

**Affiliations:** 1 Department of Public Health Sciences, Henry Ford Health, 48202 Detroit, MI USA; 2 Department of Obstetrics, Gynecology, and Reproductive Biology, College of Human Medicine, Michigan State University, 965 Wilson Rd, 48224 East Lansing, MI USA; 3 Department of Obstetrics and Gynecology, Henry Ford Health, 48202 Detroit, MI USA

**Keywords:** Racial disparities, Myomectomy, Area Deprivation, Financial toxicity

## Abstract

**Background:**

At time of myomectomy, a surgical procedure to remove uterine fibroids, Black women tend to have larger uteri than White women. This makes Black patients less likely to undergo a minimally invasive myomectomy which has been shown to have less postoperative pain, less frequent postoperative fever and shorter length of stay compared to abdominal myomectomies. The associations between individual financial toxicity and community area deprivation and uterine volume at the time of myomectomy have not been investigated.

**Methods:**

We conducted a secondary data analysis of patients with fibroids scheduled for myomectomy using data from a fibroid treatment registry in [location]. We used validated measures of individual-level Financial Toxicity (higher scores = better financial status) and community-level Area Deprivation (ADI, high scores = worse deprivation). To examine associations with log transformed uterine volume, we used linear regression clustered on race (Black vs. White).

**Results:**

Black participants had worse financial toxicity, greater deprivation and larger uterine volumes compared with White participants. A greater Financial Toxicity score (better financial status) was associated with lower uterine volume. For every 10 unit increase in Financial Toxicity, the mean total uterine volume decreased by 9.95% (Confidence Interval [CI]: -9.95%, -3.99%). ADI was also associated with uterine volume. A single unit increase in ADI (worse deprivation) was associated with a 5.13% (CI: 2.02%, 7.25%) increase in mean uterine volume.

**Conclusion:**

Disproportionately worse Financial Toxicity and ADI among Black patients is likely due to structural racism – which now must be considered in gynecologic research and practice.

**Trial Registration:**

Not applicable.

**Supplementary Information:**

The online version contains supplementary material available at 10.1186/s12905-023-02761-x.

## Introduction

Uterine leiomyomas (fibroids) are benign uterine neoplasms that commonly cause abnormal uterine bleeding and bulk symptoms that can include pelvic pain, urinary frequency and constipation [1]. There are various racial disparities in the incidence and treatment of fibroids. Approximately 70% of White women and more than 80% of Black women will have had fibroids by the time they reach menopause [2]. At the time of treatment, Black women tend to have more and larger fibroids than White women [3–6]. Black women are more likely than White women to schedule uterine-sparing treatments (e.g., myomectomy and uterine artery embolization vs. hysterectomy – the removal of the uterus) [7].

Myomectomy is a uterine-sparing treatment in which fibroids are “shelled out” of the uterus either laparoscopically, through small minimally invasive incisions, or through a laparotomy, which requires an open vertical or horizontal incision across the abdomen. Patients with very large uteri are less likely to have a minimally invasive myomectomy compared with patients with smaller uteri. Minimally invasive myomectomy has been shown to have less postoperative pain, less frequent postoperative fever and shorter length of stay in the hospital compared with all abdominal myomectomies [8]. Black patients tend to have larger uteri than White patients at the time of fibroid treatment and these larger uteri could decrease the patients’ likelihood of experiencing the benefits of minimally invasive myomectomy.

We do not know why Black women tend to have larger uteri at the time of fibroid treatment. When, how, and why women seek care for their fibroids is not well understood. Half of women with fibroids are undiagnosed [9] even though many women with undiagnosed fibroids report having fibroid symptoms [10]. Anecdotally, we have observed that women having a myomectomy at our urban hospital, Henry Ford Hospital in Detroit, MI, have larger uteri at the time of surgery compared with women having surgery at our suburban hospital, Henry Ford West Bloomfield Hospital. The patient populations differ across these hospitals with respect to socioeconomic factors that could influence the patients’ abilities to seek care. We investigated whether these factors were associated with uterine size at time of treatment. More specifically, using data from participants in the Comparing Options for Management: PAtient-Centered REsults for Uterine Fibroids (COMPARE-UF) fibroid treatment registry enrolled at Henry Ford Health (HFH) who had a myomectomy, we examined the associations between Financial Toxicity, an individual-level measure of financial distress related to medical treatment [11], and the Area Deprivation Index (ADI) [12, 13], a community-level measure that is a composite score of 17 indicators of socioeconomic disadvantage, and uterine volume calculated from imaging details abstracted from participant medical records [12, 14]. We are just beginning to investigate how stress may affect fibroid formation and growth and area-based community-level factors are an important stress pathway [15, 16]. Understanding an individual’s and an area’s socioeconomic status may improve care, for example, access to treatments has been shown to be limited due to high procedural costs [17]. Further, including individual and composite proxy measures of area socioeconomic status into the electronic medical record is increasingly being implemented to improve quality of care [17–19].

## Materials and methods

We conducted a secondary analysis using data from a single longitudinal cohort, COMPARE-UF registry site HFH in Metropolitan-Detroit, Michigan. COMPARE-UF is a multi-site registry across the United States comprised of eight clinical sites and two additional UAE specialty clinics. Women scheduled for a fibroid treatment from January 2017 through December 2019 were invited to complete a baseline survey that included validated measures of quality of life, financial toxicity, mental health, and sociodemographic and reproductive history [14, 20–23]. As part of COMPARE-UF protocols, chart review was conducted for each participant’s treatment at baseline, including treatment details and uterine and fibroid characteristics. Uterine dimensions were taken from the participant ultrasounds. Eligible women were 18–55 years of age at the time of fibroid treatment. Full study details have been described previously [7, 24–27]. Black and White women that had a myomectomy by any surgical approach were included in these analyses. Analyses were limited to those patients who had a myomectomy because uterine size can affect whether or not the patient may have a minimally invasive surgical approach. Uterine size does not affect the approaches of other uterine-preserving fibroid treatments. This study was approved by the local institutional review board and all participants provided informed consent.

The dependent variable for these analyses was uterine volume (cm^3^). Uterine volume was measured by multiplying the maximum length, anteroposterior, and transverse diameters of the uterus, as estimated by ultrasound, before multiplying by 0.52 to estimate a prolate ellipsoid [28]. The dependent variable was log transformed to improve model fit.

The independent variables were financial toxicity and area deprivation [13, 14, 29, 30]. Financial toxicity was measured using the COmprehensive Score for Financial Toxicity (COST) scale. COST is a self-reported outcome measure that describes financial distress. Participants are asked to answer 12 questions on a five-point Likert scale from “not at all” (0) to “very much” (4) as it applies to the previous seven days. Questions include “I know I have enough money in savings, retirement, or assets to cover the costs of my treatment” and “I am concerned about keeping my job and income, including work at home”. Higher scores indicate better financial well-being (i.e., less toxicity). Area deprivation was measured using the ADI available through the Neighborhood Atlas, from the University of Wisconsin-Madison School of Medicine and Public Health. The ADI is validated and allows for ranking of neighborhoods by socioeconomic disadvantage down to the census block group level using American Community Survey 5-year data. It includes a linear combination of 17 US Census indicators of income, education, employment, and housing quality (Supplemental Table 1) [30, 31]. Area-level composite measures of socioeconomic status, such as the ADI, are typically considered more robust [32]. The ADI for this study was measured by mapping individual-level address data to census block groups using publicly available ADI deciles through ArcGIS [33]. The ADI is available within a state as deciles from one to ten. Higher scores on the ADI indicate more disadvantage.

We used counts and percentages and means and standard deviations to describe the study population overall and by race. Age (continuous and categorical, years), ethnicity (Hispanic vs. non-Hispanic), insurance type (private vs. other, which includes Medicaid, Medicare, uninsured, and “other” categories), relationship status, education level, body mass index (BMI; kg/m^2^), previously pregnant (yes vs. no), number of fibroids measured (continuous), financial toxicity, uterine volume (cm^3^), and area deprivation. To compare distributions of these variables between Black and White racialized groups, we used Pearson chi-square tests and the Wilcoxon rank-sum statistics for categorical variables and continuous variables, respectively.

We used unadjusted linear regression clustered on patient race to estimate associations and 95% confidence intervals (CI). Clustering is a robust regression method and allows for correction of standard error estimates when a statistical model assumption might be violated (e.g., variance-covariance matrix), by “relaxing” the assumption of independence [34]. All analyses were performed using Stata SE 17 [35]. We chose to cluster on race as we believed that patients who are Black are more similar to other patients who are Black and patients who are White are more similar to other patients who are White with respect to ADI and Financial Toxicity and uterine volume.

## Results

**Fig. 1:**
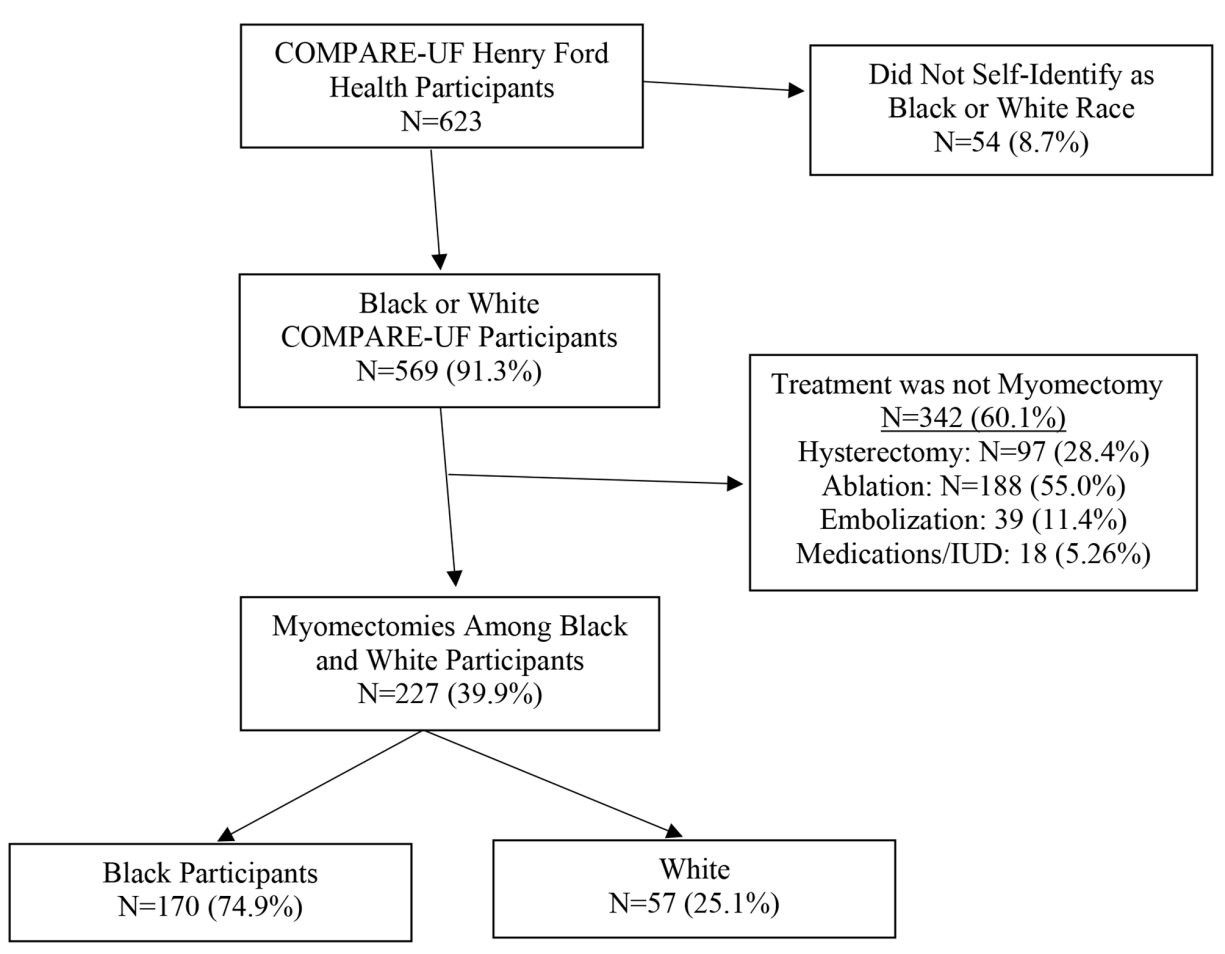
Derivation of the Analytical Population from all Henry Ford Health COMPARE-UF Participants

**Fig. 2:**
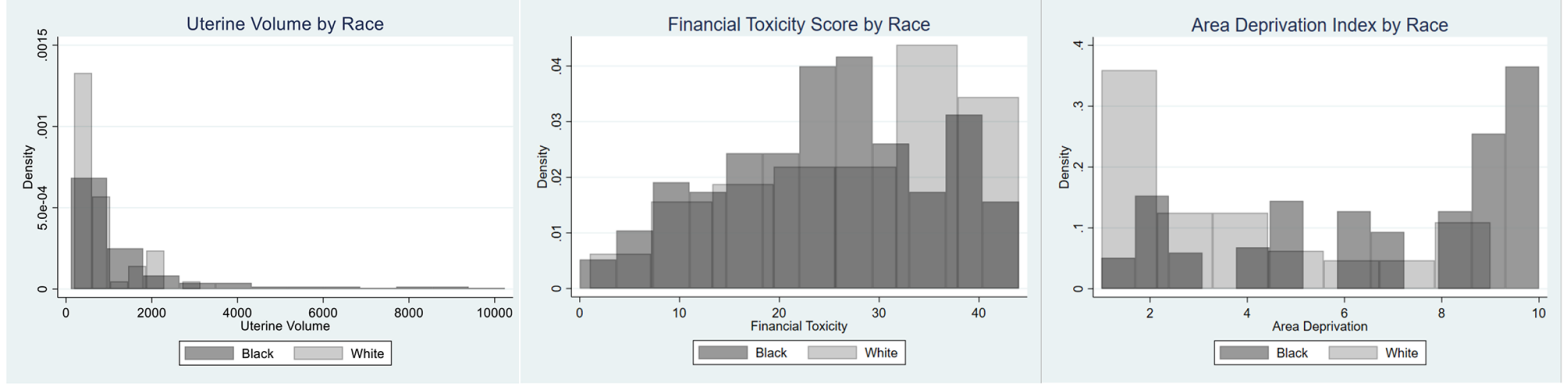
The Distribution of Total Uterine Volume (cm^3^) by Race (left). X Axis is Uterine Volume (cm^3^). The Distribution of Financial Toxicity Score by Race (middle). The Distribution of Area Deprivation Index by Race (right). Y Axes are Density of Participants. Black Participants are in Dark Gray and White Participants are in Light Gray

Among the 623 COMPARE-UF participants, there were 227 participants who had a myomectomy and self-identified as either Black (n = 170) or White (n = 57). (Fig. 1) The groups were similar in average age and educational status. (Table 1) The average age of participants was 39.0 and 40.3 for Black women and White women, respectively. The percentage of self-reported Non-Hispanic ethnicity was also similar for Black (98.2%) and White participants (96.5%). Black women were less likely to have private insurance (74.7% vs. 86.0%), be married (43.1% vs. 53.6%), and more likely to have a prior pregnancy (71.4% vs. 56.1%) and a higher BMI (32.9 vs. 28.6 kg/m2) compared with White women. Black women had tended to have more fibroids and greater total uterine volume at time of myomectomy. (Fig. 2) Overall, White women had higher financial toxicity scores indicating less disadvantage (median (IQR) for White women = 27.8 (11.1) and Black women = 24.6 (10.9); p < 0.028) and lower ADI (median (IQR) for White women = 3.0 (3.0) and Black women = 8.0 (5.0); p < 0.05) compared with Black women. (Fig. 2)

**Table 1:** Baseline Characteristics by Race among Henry Ford Health Myomectomy Patients in the COMPARE-UF Study, N (%) Unless Other Statistic Noted in Row

	Total Participants (N = 227)		Black Participants (N = 170)		White Participants (N = 57)		
	N	%	N	%	N	%	*p*-value
Age (Years), median (IQR)	38.9 (10.2)		38.9 (10.1)		38.9 (11.4)		0.25
Age (Years)							0.41
<=34	66	29.1	51	30.0	15	26.3	
35–39	63	27.8	46	27.1	17	29.8	
40–44	47	20.7	39	22.9	8	14.0	
45–49	32	14.1	22	12.9	10	17.5	
50–54	19	8.4	12	7.1	7	12.3	
Non-Hispanic	222	97.8	167	98.2	55	96.5	0.44
Insurance Type							0.08
Private	176	77.5	127	74.7	49	86.0	
Other	51	22.5	43	25.3	8	14.0	
Relationship Status^NA=4^							< 0.05
Married	102	45.7	72	43.1	30	53.6	
Separated	4	1.8	4	2.4	0	0.0	
Divorced	25	11.2	11	6.6	14	25.0	
Widowed	5	2.2	4	2.4	1	1.8	
Single	87	39.0	76	45.5	11	19.6	
Education Level^NA=1^							0.32
Less than High School	4	1.8	3	1.8	1	1.8	
High School Graduate	33	14.6	27	16.0	6	10.5	
Some College	87	38.5	66	39.1	21	36.8	
College Graduate	56	24.8	44	26.0	12	21.1	
Graduate Degree	46	20.4	29	17.2	17	29.8	
Body Mass Index (kg/m^2^), median (IQR)^NA=5^	29.9 (10.2)		30.3 (10.7)		28.7 (7.59)		< 0.01
Previously Pregnant^NA=2^	152	67.6	120	71.4	32	56.1	< 0.05
Number of Fibroids Measured, median (IQR)^NA=12^	1.0 (2.0)		2.0 (2.0)		1.0 (1.0)		< 0.01
Number of Fibroids Measured							< 0.05
1	112	52.1	77	47.0	35	68.6	
2	40	18.6	32	19.5	8	15.7	
3	29	13.5	26	15.9	3	5.9	
4+	34	15.8	29	17.7	5	9.8	
Minimally Invasive Myomectomy	166	73.1	117	68.8	49	86.0	
Uterine Volume (cm^3^), median (IQR)^NA=21^	682.8 (1102.3)		771.1 (1220.1)		576.6 (519.5)		< 0.001
Financial Toxicity, median (IQR)^NA=18, a^	25.4 (11.0)		24.6 (10.9)		27.8 (11.1)		< 0.028
Area Deprivation Index, median (IQR)^NA=1, b^	6.0 (6.0)		8.0 (5.0)		3.0 (3.0)		< 0.05

“Other” Insurance Type: includes Medicaid, Medicare, Uninsured, and “Other”

NA: Missing

IQR: Interquartile Range

Only the first four fibroid dimensions were present, or sample size was too small by race

^a^ Measured by COmprehensive Score for financial Toxicity (COST) (higher score = better). Possible score range is 0–44

^b^ADI: Measured by the Area Deprivation Index (Higher score = worse). Possible score range is 0–10

N = 1 Suppression due to a high group quarters population, grouped into intuitional (e.g., correctional facilities, nursing homes) and non-institutional (e.g., military barracks, shelters) [1–3]

In the linear regression model of log transformed uterine volume, greater financial toxicity score (i.e., less financial burden) was associated with lower uterine volume (Table 2). For a 10 unit increase in financial toxicity, the mean total uterine volume decreased by 9.95%. Separately, ADI was associated with uterine volume (Table 2). A single unit increase in ADI was associated with a 5.13% increase in mean uterine volume (Table 2).

**Table 2:** Financial Toxicity and Area Deprivation Index (ADI) and Log Transformed Uterine Volume, Linear Regression with Clustering on Race (Black or White)

	Uterine Volume (log cm^3^)					
	Beta	SE	95% CI	Percent Change in the mean total uterine volume for a 10 unit change in the Financial Toxicity Score and ADI^a^	Percent Change 95% CI	*p*-value
Financial Toxicity^b^	-0.01	0.002	-0.01, -0.01	-9.95%	-9.95%, -3.99%	< 0.001
ADI^c^	0.05	0.01	0.02, 0.07	5.13%	2.02%, 7.25%	< 0.001

SE: Standard error

CI: Confidence Interval

^a^100*e^beta^-1 is the percent change in the mean total uterine volume for a 10 unit change in Financial Toxicity

^b^Measured by COmprehensive Score for financial Toxicity (COST) (higher score = better financial status)

^c^ADI: Measured by the Area Deprivation Index (Higher score = worse deprivation)

## Discussion

In this study of myomectomy patients at a single health system, Black participants had larger uterine volumes, were more likely to report worse financial status and lived in areas with greater deprivation at the time of myomectomy compared with White participants. Financial status and area deprivation were also both associated with uterine volume. Based on these results, we hypothesize that structural racism may lead to disproportionately greater area deprivation and worse financial status for Black compared with White individuals. In turn, these factors create barriers to receiving timely care as women can sometimes view their health issues as secondary when faced with financial or other challenges, even when symptomatic. This delay in obtaining medical care subsequently limits minimally invasive treatment options available to Black women as a result of larger uteri at time of surgery.

ADI and Financial Toxicity may also represent levels of chemical and non-chemical stressors in the communities of participants that may affect the growth of their fibroids through epigenetic mechanisms. For example, in Eker rat models, early-life exposure to endocrine disrupting chemicals (e.g., diethylstilbestrol) have been shown to increase fibroid penetrance, number, and size [36–39]. Further gene mutations, MED12 and HMGA2 with epigenetic modification by TET3, have been found to contribute to fibroid risk, size, and subtype. [40, 41] Additionally, promoter DNA methylation-mediated gene silencing may contribute to fibroid etiology in Black Women [42]. Disparities in exposure to endocrine disrupting chemicals through racism and segregation may have resulted in observed differences in UF by race, income, and neighborhood [43–48].

We also think that understanding a patient’s financial situation and residential environment are important components of care and could become part of the Meaningful Use data that are routinely collected during the patient intake and rooming process. Literature suggests that socioeconomic indicators are good indicators to affect patient outcomes and reduce health disparities by addressing barriers. For example, provider knowledge about financial toxicity may prompt conversations about lower priced prescriptions [49].

In analyses of COMPARE-UF participants across all sites, Black participants who scheduled a hysterectomy had greater uterine volumes and worse financial toxicity scores than White participants, but this was not true for participants scheduled for uterine artery embolization (UAE) [7]. This difference could reflect that historically, UAE was not recommended for larger uteri [50]. The relationships between financial toxicity and area deprivation and uterine size have not yet been investigated in these treatment groups. There were too few participants at our site who had a UAE and therefore we did not analyze those data. We are not aware of other studies of fibroid treatments that have assessed self-reported financial impact of treatment with a standardized questionnaire or examined the role of area deprivation for the patients.

Others have also reported on the racial differences in uterine size or fibroid characteristics at time of diagnosis or treatment. In analyses of data from chart reviews of 386 women who had a myomectomy at a large academic center, African American women (31%; n = 121) had more fibroids than White women as determined by both preoperative imaging (African American women: 36% with 3 or more myomas; White women: 19% with 3 or more myomas; p < 0.05) and by operative report (> 8 fibroids: 31% versus 13%; p < 0.05) [3]. In patients scheduled for a hysterectomy or myomectomy at George Washington University, Black women had bigger uteri than White women [4]. These studies did not identify sources of the disparities in uterine volume. Importantly, among Black people financial toxicity and ADI have been associated with other adverse outcomes. For example, worse financial toxicity has been associated with risk of triple negative breast cancer and gynecologic cancer; [51–53] worse ADI has been associated with breast cancer and cardiovascular mortality compared with White people [54, 55], indicating that the effect of individual and neighborhood-level toxicity and deprivation may have far reaching effects on health for Black populations.

Results of these analyses may not be generalizable to other regions or patient populations. Unfortunately, we did not ask participants to report barriers to care such as transportation and the ability to take time away from work or other care duties. Although this work is not causative, we do think the data presented here provide support for this ongoing area of research. For example, race is a social construct, does not represent a biological variable, and through mechanisms such as structural racism has contributed to racialized health disparities [56]. In particular, historically imposed marginalization (e.g., slavery) and individual-level assignment to racial groups by phenotypic expression created contemporaneous factors (e.g., redlining) that have shaped physical, economic, social, and neighborhood contexts at the individual (e.g., financial toxicity) and ecological-level (e.g., area deprivation) that effect health-related factors (e.g., uterine volume) [56].

## Conclusions

We propose that it is time to stop cataloging these racialized differences and begin to conduct the challenging work of identifying their sources. We think our results highlight the importance of including patient and community level factors in understanding why Black patients have larger uteri at the time of treatment. However, understanding the role of structural racism in disease development and treatment-seeking and treatment-providing activities is essential. This is of importance because patients with larger uteri, a group that is disproportionately comprised of Black individuals, are less likely to have a minimally invasive myomectomy and benefit from its faster recovery time and quicker return to work further contributing to their financial toxicity.

### Electronic supplementary material

Below is the link to the electronic supplementary material.

Supplementary Material 1

## Data Availability

The deidentified data that support the findings of this study are available on request from the corresponding author ASB with appropriate data use agreements. The data are not publicly available due to state restrictions e.g., data contains information that could compromise research participant privacy/consent.

## Abbreviations

**ADI**: Area Deprivation
**COMPARE-UF**: Comparing Options for Management:Patient-Centered Results for Uterine Fibroids
**HFH**: Henry Ford Health
**COST**: Comprehensive Score for Financial Toxicity scale
**BMI**: Body Mass Index
**CI**: Confidence Interval
**SD**: Standard Deviation
**UAE**: Uterine Artery Embolization
