# Supplementary material for: Racial disparities between measures of area deprivation and financial toxicity, and uterine volume in myomectomy patients
Source: BMC Womens Health. 2023 Nov 14;23:603. doi: 10.1186/s12905-023-02761-x (PMC10648622; doi:10.1186/s12905-023-02761-x)
Supplement: Supplementary file 1 — Supplementary Material 1 [file 12905_2023_2761_MOESM1_ESM.docx]

Supplemental Table 1

Percent of population aged >= 25 years with < 9 years of education

Percent of population aged >= 25 years with < a high school diploma

Percent of employed persons >=16 years of age in white-collar occupations

Median family income

Income disparity (Defined as the log of 100 * the ratio of the number of households with <$10,000 in income to the number of households with $50,000 or more in income.)

Median home value

Median gross rent

Median monthly mortgage

Percent owner-occupied housing units (home ownership rate)

Percent of civilian labor force population >= 16 years of age unemployed (unemployment rate)

Percent of families below the poverty level

Percent of population below 150% of the poverty threshold

Percent of single-parent households with children < 18 years of age

Percent of households without a motor vehicle

Percent of households without a telephone

Percent of occupied housing units without complete plumbing

Percent of households with more than one person per room (crowding).
